# The association of TNF-alpha secretion and mtDNA copy number in CD14^+^ monocytes of patients with obesity and CHD

**DOI:** 10.3389/fmolb.2024.1362955

**Published:** 2024-03-20

**Authors:** Taisiya V. Tolstik, Tatiana V. Kirichenko, Alexander M. Markin, Anastasia I. Bogatyreva, Yuliya V. Markina, Diana G. Kiseleva, Nataliya N. Shaposhnikova, Antonina V. Starodubova, Alexander N. Orekhov

**Affiliations:** ^1^ Petrovsky National Research Center of Surgery, Moscow, Russia; ^2^ Chazov National Medical Research Center of Cardiology, Moscow, Russia; ^3^ Рeoples’ Friendship University of Russia Named After Patrice Lumumba (RUDN University), Moscow, Russia; ^4^ Department of Biophysics, Faculty of Biology, Lomonosov Moscow State University, Moscow, Russia; ^5^ Federal Research Centre for Nutrition, Biotechnology and Food Safety, Moscow, Russia

**Keywords:** CD14^+^ monocytes, primary culture, TNF-α, mitochondrial DNA copy number, obesity, coronary heart disease

## Abstract

**Introduction:**

Mitochondrial dysfunction may be one of the causes of inflammatory activation of monocytes and macrophages, which leads to excessive secretion of inflammatory mediators and the development of chronic inflammation.

**Aims:**

The study was aimed to evaluate the secretion of inflammatory cytokine tumor necrosis factor-α (TNF-α) in the primary culture of monocytes, and to analyze its relationship with the number of mitochondrial DNA (mtDNA) copies in the blood of patients with coronary heart disease (CHD) and obesity.

**Materials and methods:**

108 patients with obesity and concomitant CHD and a control group of 25 participants were included in the study. CD14^+^ monocytes were isolated by a standard method in a ficoll-urographin gradient, followed by separation using magnetic particles. The number of mtDNA copies was estimated using qPCR.

**Results:**

It was demonstrated that the number of mtDNA copies was significantly increased in groups of patients with CHD and obesity + CHD in comparison with control group. mtDNA copy number positively correlated with basal and LPS-stimulated TNF-α secretion, the most significant correlation was found in the group of patients with CHD and obesity.

**Conclusion:**

Thus, the change in mtDNA copy number in CD14^+^ monocytes which indicates the presence of mitochondrial dysfunction, confirm the direct involvement of mitochondria in the violation of the inflammatory response of monocytes revealed in this study as an increased secretion of inflammatory cytokine TNF-α.

## Introduction

Coronary heart disease (CHD) is a widespread dangerous cardiovascular disease, leading to an increase in mortality and disability worldwide (Shaya et al., 2022). CHD is caused by a reduction in blood supply in the myocardium due to atherosclerotic stenosis of the coronary arteries (Polimeni, 2020). A number of risk factors contribute to the development of CHD, including smoking, elevated blood cholesterol levels, arterial hypertension, diabetes mellitus, overweight or obesity (AlZaim et al., 2023), physical inactivity, and hereditary predisposition (Malakar et al., 2019). At present, it has been convincingly proven that inflammatory processes in the arterial wall contribute to the development and progression of atherosclerotic lesions, which are of key importance in the pathogenesis of CHD (Liu et al., 2021; Sagris et al., 2021). Due to cholesterol accumulation in the arterial wall, inflammatory cells, in particular, monocytes, are recruited into the intima of arteries, activated under the influence of stimulating factors with further differentiation into macrophages. In the arterial wall, these cells secrete cytokines (eg, interleukin-1, interleukin-6, tumor necrosis factor-α (TNF-α)) and other inflammatory mediators, which increase the inflammation and lead to endothelial damage, activation of smooth muscle cells, and further formation of atherosclerotic plaques (Song et al., 2022). The inflammatory process also contributes to the destabilization of atherosclerotic plaques, which can cause their rupture and clots formation (Susser and Rayner, 2022).

Obesity is one of the main risk factors for the development of CHD (Jensen et al., 2020). Overweight and obesity are associated with a number of pathological processes in the organism, in particular, with dyslipidemia and increased blood pressure (AlZaim et al., 2022), which contribute to the development of atherosclerosis and increase the risk of CHD development (Kojta et al., 2020). There are a number of studies demonstrating that inflammation plays a significant role in the pathogenesis of obesity. Adipose tissue actively secretes inflammatory mediators such as cytokines, chemokines, and growth factors (Ying et al., 2019). Chronic inflammation observed in obesity causes a violation of metabolic processes due to more active metabolism in adipocytes, which produce cytokines and other inflammatory factors (Ahmed et al., 2021). Inflammatory mediators’ production in adipose tissue is a key pathogenetic mechanism of insulin resistance development, that leads to the development of type 2 diabetes, which is often associated with obesity. Inflammation can also affect the formation and activation of adipocytes, that leads to disruption of the mechanisms of regulation of energy balance and appetite (van Baak and Mariman, 2019; Alzaim et al., 2020).

Monocytes are key cells in the pathogenesis of inflammation. Monocytes migrating from the blood to the tissues where the inflammatory process occurs, differentiate into activated macrophages, which are involved in all stages of the pathogenesis of chronic inflammation (Amengual and Barrett, 2019). Macrophages eliminate microorganisms, infectious agents and other pathogenic substances, secrete cytokines that regulate the inflammatory response in the organism activating other cells and tissues (Halade and Lee, 2022). Mitochondrial dysfunction may be one of the causes of inflammatory activation of monocytes and macrophages, which leads to violation of the inflammatory response, over secretion of inflammatory mediators, and the development of chronic inflammation (Luan et al., 2021). TNF-α is one of the inflammatory cytokines secreted by macrophages, that plays an important role in the pathogenesis of atherosclerosis and obesity (Kirichenko et al., 2022). It is able to activate cells of the immune system, and is also involved in the regulation of apoptosis (Henein et al., 2022). TNF-α stimulates the expression of adhesion molecules on the surface of endothelial cells, which helps to regulate the migration of monocytes to the site of inflammation (Indumathi et al., 2022). Moreover, TNF-α induces the secretion of metalloproteinases (MMPs) by macrophages under the control of microRNAs. MMPs catalyze the destruction of interstitial collagen, which leads to thinning and weakening of the fibrous capsule, thereby making the plaque unstable and increasing the risk of plaque rupture and thrombosis (Almassabi et al., 2023).

Mitochondria are known to be sources of damage-associated molecular fragments (damps) such as ATP, formyl peptide, and mitochondrial DNA (mtDNA). An important role of mtDNA in the initiation of inflammation is that it is released from damaged mitochondria and activates pattern recognition receptors (PRRS), including toll-like receptor 9 and cytosolic infammasomes (Ye et al., 2023). It was shown that patients with chronic metabolic diseases had an increased amount of circulating mitochondrial DNA compared to healthy people, which positively correlated with the concentration of TNF-α in the blood serum (Deng et al., 2020). In addition, the changes in the number of circulating mtDNA copies were found in patients with obesity and hypertension. It was demonstrated that monocyte stimulation with mtDNA led to increase of TNF-α secretion, which suggests that mtDNA can modulate the production of proinflammatory cytokines (Pinti et al., 2014).

Currently inflammatory response of innate immune cells including monocytes is widely investigated in pathogenesis of cardiovascular and other chronic diseases (Bahrar et al., 2024). Mitochondrial dysfunction in monocytes may be considered as a potential mechanism of the violation of their inflammatory status (Orekhov et al., 2020). In current study mtDNA copy number was used as a surrogate marker of mitochondrial dysfunction since it was demonstrated in several studies that mt DNA copy number strongly associated with some indicators of mitochondrial function such as oxidative stress parameters, energy reserves, and mitochondrial membrane potential (Castellani et al., 2020). The results of previous clinical studies allow considering mtDNA copy number as a potential diagnostic and prognostic marker in CHD and other chronic diseases (Li et al., 2022). The aim of this study was to evaluate the association of changes in mitochondrial DNA copy number and violation of inflammatory response of circulating monocytes considered as excess secretion of inflammatory cytokine TNF-α in primary culture of monocytes obtained from patients with CHD and obesity.

## Subjects and methods

### Study design

The pilot cross-sectional study included men and women aged from 50 to 75 years in four groups:

1) healthy individuals with a normal body mass index (BMI) from 18 kg/m2 to 25 kg/m^2^ without a history of CHD;
2) patients with obesity, BMI>30 kg/m^2^, without a history of CHD;
3) patients with a normal BMI from 18 kg/m2 to 25 kg/m2 with a history of CHD;
4) patients with obesity, BMI>30 kg/m^2^, and a history of CHD.

The exclusion criteria were age under 50 or over 75 years old, as well as the presence of severe chronic diseases and comorbid conditions that could affect the results of the assessment, such as diabetes mellitus, chronic renal failure, chronic liver failure and cancer. This work was approved by the Ethics Committee of the Federal Research Centre for Nutrition, Biotechnology and Food Safety (protocol #3, at 13 December 2021). All study participants signed informed consent to participate in the study.

### Primary culture of monocytes

The study model was a primary culture of human CD14^+^ monocytes obtained from 30 mL of whole blood from all study participants. A mononuclear cells fraction was obtained by centrifugation using a ficoll solution (PanEco, Russia, P053) with a density of 1.077 g/cm3. Then the cells were resuspended in buffer optimized for use in the MACS magnetic cell sorting system and a fraction of CD14^+^ monocytes was obtained using paramagnetic nanoparticles CD14 MicroBeads (Miltenyi Biotec, USA, 130-050-201) using magnetic separation columns (Miltenyi Biotec, USA, 130-042-201). CD14 was used as a specific marker of monocytes/macrophages (Ziegler-Heitbrock and Ulevitch, 1993). The isolated cells were cultured in X-Vivo 10 medium (Lonza, Germany, 04-380q) containing L-glutamine, gentamicin, and phenol red in two wells of a 48-well plate at a density of 500,000 cells per well in a CO_2_ incubator at 37°C (with 95% air and 5% CO2) for 7 days. The culture media from the first well was used to determine the basal secretion of cytokines after 24 h of cultivation. In the second well, the inflammatory response of monocytes was stimulated using lipopolysaccharide (LPS). LPS is a universal agent widely used for pro-inflammatory stimulation of monocytes/macrophages in *ex vivo* models, and therefore it was used in the current study to investigate the immune response of monocytes in chronic inflammatory conditions such as CHD and obesity (Page et al., 2022). The culture media from the second well was used to determine LPS-stimulated secretion in 24 h after the first stimulation, then the culture media was changed, the cells were cultured for 5 days without inflammatory stimulation. On the sixth day of the experiment, the culture media was changed again and the cells were re-stimulated with LPS. On the seventh day, in 24 h after the second stimulation, culture media samples were obtained to assess the tolerance of the immune response of cells to repeated inflammatory stimulation. The supernatant samples were stored in a freezer at −70°C until the level of TNF-α secretion was determined using Human TNF-alpha/TNFSF1A DuoSet ELISA kits (R&D Systems, USA, EH0302).

### Mitochondrial DNA copy number

DNA was isolated by phenol-chloroform extraction from blood-derived CD14^+^ monocytes obtained at the previous stage of the experiment (Köchl et al., 2005). The concentration and quality of the isolated DNA was evaluated using a spectrophotometer (NanoPhotometer, Implen GmbH, Germany). The quantitative real-time polymerase chain reaction (PCR) was performed for mitochondrial DNA copies determination. The measurement of mitochondrial DNA (mtDNA) copy number was conducted similarly to the previously described method (Venegas and Halberg, 2012; Gu et al., 2013). For this purpose, 2 pairs of primers were selected: for the mitochondrial MTND2 gene, as well as for the nuclear β-2-microglobulin gene. The ND4 gene was used to represent the mtDNA and the NCOA3 gene was used to represent the nDNA.

The sequence of primers for ND4: forward 5′-CCA​TTC​TCC​TCC​TAT​CCC​TCA​AC-3′; reverse 5′-CAC​AAT​CTG​ATG​TTT​TGG​TTA​AAC​TAT​ATT​T-3′. The sequence of primers for NCOA3: forward 5′-GAG-TTT-CCT-GGA-CAA-ATG-AG-3’; reverse 5′-CAT-TGT-TTC-ATA-TCT-CTG-GCG-3’.

### Flow cytometry

The CD14^+^ monocyte population was analyzed using flow cytometry on a MACSQuant VYB flow cytometer (Miltenyi Biotec, Germany) with antibodies to the CD 14 marker labeled with phycoerythrin (Cusabio Biotech, China, CSB-MA804053) and CD14 antibodies labeled with FITC (Cusabio Biotech, China, F084401). The cells were resuspended in a PBS buffer at the rate of 10^5^ cells per 100 µL. 5 μL of antibodies at a concentration of 100 mM were added to the resulting suspension. The samples were incubated for 40 min at room temperature in the dark. After incubation, the cells were washed 3 times in 500 µL of PBS buffer. .

### Statistical analysis

Statistical analysis of the obtained data was carried out using the R statistics software (version 2023.03.1 + 446). The Shapiro-Wilk W test was used to test the type of distribution. For a comparative analysis of data in the studied groups of patients with the control group, the Mann-Whitney U-test was used to analyze the results obtained between all groups, and the Dunn test was additionally used to check the results for statistically significant differences between groups. When analyzing the number of mtDNA copies per cell, the abnormal distribution was found, so further analysis was performed using the Kruskal-Wallis test. Differences were considered statistically significant at *p* < 0.05. Data are presented as median and quartiles (Me [Q1; Q3]).

## Results

### Characteristics of study participants

A total of 124 participants were included into 4 groups:

1) control group of healthy volunteers, n = 25;
2) obesity group of CHD - free participants with obesity, n = 39;
3) CHD group of normal weight patients with CHD, n = 29;
4) obesity + CHD group of patients with obesity and CHD, n = 40.

Table 1 presents the clinical and laboratory characteristics of study participants.

**TABLE 1:** Clinical and laboratory characteristics of study participants.

Characteristics	Control	Obesity	CHD	Obesity + CHD
Age, years	63 [54; 68]	61 [63; 69] *p* > 0.05	68 [65; 71] *p* > 0.05	64 [59; 68] *p* > 0.05
Gender, f/m	18/7	29/10 *p* > 0.05	11/9 *p* > 0.05	27/16 *p* > 0.05
SBP, mmHg	130 [128; 132]	132 [130; 136] *p* > 0.05	129 [120; 139] *p* > 0.05	126 [123; 128] *p* > 0.05
DBP, mmHg	78 [75; 80]	80 [80; 86] *p* < 0.05	78 [72; 80] *p* < 0.05	75 [74; 79] *p* < 0.05
Arterial hypertension, %	26.9	66.6 *p* < 0.001	95 *p* < 0.001	97 *p* < 0.001
BMI, kg/m^2^	23.3 [22.6; 25.5]	38.8 [34.8; 41.9] *p* < 0.001	24.1 [23; 25.6] *p* > 0.05	40.7 [35.3; 45.5] *p* < 0.001
Statin administration, %	14	29 *p* > 0.05	53 *p* < 0.05	72 *p* < 0.001
Total cholesterol, mg/dl	208.7 [183.3; 238.3]	197.9 [169.8; 224.1] *p* > 0.05	210.6 [174.8; 237.2] *p* > 0.05	194.1 [141.7; 222.9] *p* < 0.05
Triglycerides, mg/dl	103.0 [93.3; 140.5]	116.1 [98,1; 139.5] *p* > 0.05	105.3 [74.7; 135.9] *p* > 0.05	90.1 [73.9; 106.2] *p* > 0.05
HDL, mg/dl	61.2 [42.4; 123.2]	52.7 [43.1; 57.8] *p* < 0.05	52.7 [39.3; 63.5] *p* < 0.05	53.9 [43.1; 65.5] *p* < 0.05
LDL, mg/dl	57.4 [37.3; 111.7]	133.6 [105.5; 162.9] *p* < 0.05	125.5 [105.1; 145.9] *p* < 0.05	118.9 [93.2; 148.2] *p* < 0.05
Glucose, mmol/l	4.71 [4.5; 5.1]	5.13 [4.8; 5.4] *p* < 0.05	4.79 [3; 5] *p* > 0.05	5.14 [4.7; 5.7] *p* < 0.05
Blood leukocytes, 10^9^/L	7.0 [6.0; 8.1]	7.2 [6.4; 8.4] *p* > 0.05	7.0 [5.4; 8.8] *p* > 0.05	7.3 [6.3; 8.5] *p* > 0.05
Blood monocytes, 10^9^/L	0.54 [0.43; 0.65]	0.49 [0.45; 0.56] *p* > 0.05	0.54 [0.44; 0.54] *p* > 0.05	0.62 [0.49; 0.72] *p* > 0.05
cIMT, mm	0.74 [0.6; 0.78]	0.74 [0.68; 0.83] *p* > 0.05	0.8 [0.75; 0.84] *p* > 0.05	0.83 [0.75; 0.9] *p* < 0.05

Data presented as (Me [Q1; Q3]). P, difference from control group. BMI, body mass index; cIMT, carotid intima-media thickness; DBP, diastolic blood pressure; HDL, high density lipoproteins; LDL, low density lipoproteins; SBP, systolic blood pressure. The significance of difference was analyzed by Mann-Whitney U-test.

The mean age of the studied groups did not differ significantly from the control group (*p* > 0.05). When comparing the clinical and laboratory characteristics of patients, it was shown that the level of total cholesterol was significantly higher in the control group than in the obesity + CHD group (*p* < 0.05) due to statins administration by patients with obesity and CHD. The level of HDL in the control group was significantly higher than in all studied groups, and the level of LDL was significantly lower in the control group than in the obesity, CHD and obesity + CHD groups (*p* < 0.05). The value of the mean cIMT in the obesity + CHD group was significantly higher compared to the control group (*p* < 0.05).

### Immunophenotype of cultured cells

The number of CD14^+^ monocytes was expressed as a percentage of the total number of monocytes, which was taken as 100%. The population of CD14^+^ monocytes was 85%–95% of the total number of cells. CD68 was used as a macrophage marker; the percentage of CD68^+^ cells after differentiation period was 90%-95%. Cell viability was assessed using the vital dye trypan blue. The percentage of living cells after separation was 96%-98%, the percentage of living cells after incubation period was about 80%.

### TNF-α secretion in primary culture of blood derived monocytes

Basal and LPS-stimulated secretion of the inflammatory cytokine TNF-α was measured to assess the pro-inflammatory activation of circulating monocytes of all study participants. Table 2 presents the results of the measurements of TNF-α secretion in the primary culture of blood derived monocytes/macrophages.

**TABLE 2:** TNF-α secretion by cultured monocytes of study participants.

TNF-α secretion, pg/ml	Control	Obesity	CHD	Obesity + CHD
Basal secretion	78 [52; 93]	104 [85; 139] *p* < 0.05	104 [99; 177] *p* > 0.05	113 [101; 157] *p* < 0.05*
LPS-stimulated secretion	3117 [2297; 4611]	2821 [1526; 4320] *p* > 0.05	3962 [2466; 5684] *p* > 0.05	4298 [2955; 6361] *p* < 0.05*
Re-stimulated secretion	85 [88; 157]	93 [67; 116] *p* > 0.05	103 [76; 177] *p* > 0.05	125 [88; 157] *p* < 0.001**

Data presented as (Me [Q1; Q3]). P, difference from control group. The significance of difference was analyzed by Mann-Whitney U-test.

It was shown that the level of basal secretion of TNF-α in the obesity + CHD group was significantly higher than in control group of normal weight CHD-free participants. It was also shown that LPS-stimulated TNF-α secretion was significantly higher in the obesity + CHD group than in the control group and in the group of patients with obesity. In the obesity + CHD group, the level of re-stimulated secretion was also significantly higher than in the control group.

### MtDNA copy number

**FIGURE 1:**
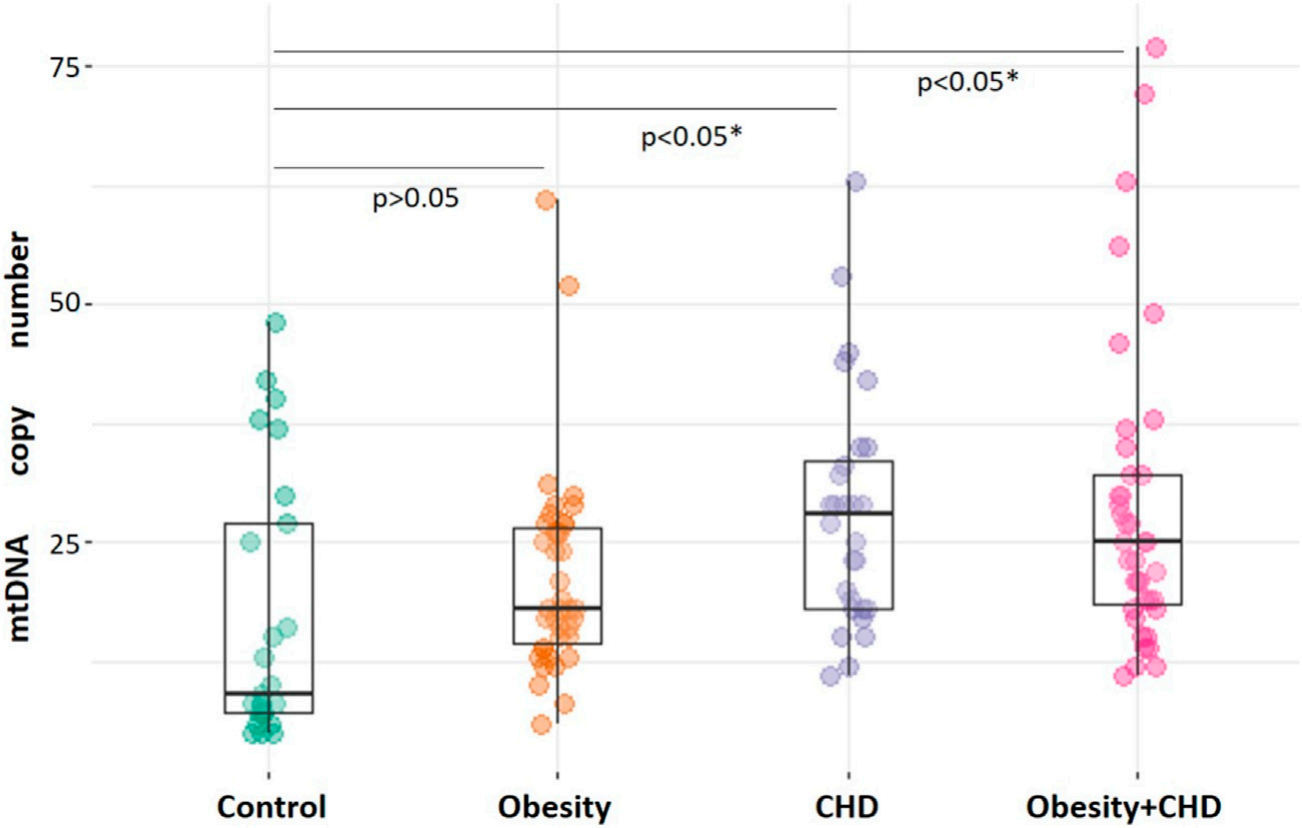
Comparative analysis of mtDNA copy number in monocytes of study participants. The significance of difference in comparison with control group is indicated. The significance of difference was analyzed by Kruskal-Wallis test.

The analysis of mtDNA copy number measurements in CD14^+^ monocytes of study participants demonstrated that the mean number of mtDNA copies in monocytes of control study participants was 18 [9; 30], that was significantly lower than mtDNA copy number in the CHD group–28 [19; 31], *p* < 0.001 and in the obesity + CHD group–25 [15; 35], *p* < 0.001. MtDNA copy number in monocytes of patients with obesity was 18 [13; 28], that was not significantly different from normal weight control study participants, *p* > 0.05. Figure 1 shows the result of comparison the number of mtDNA copies in monocytes of patients of the studied groups.

### Correlation analysis

**FIGURE 2:**
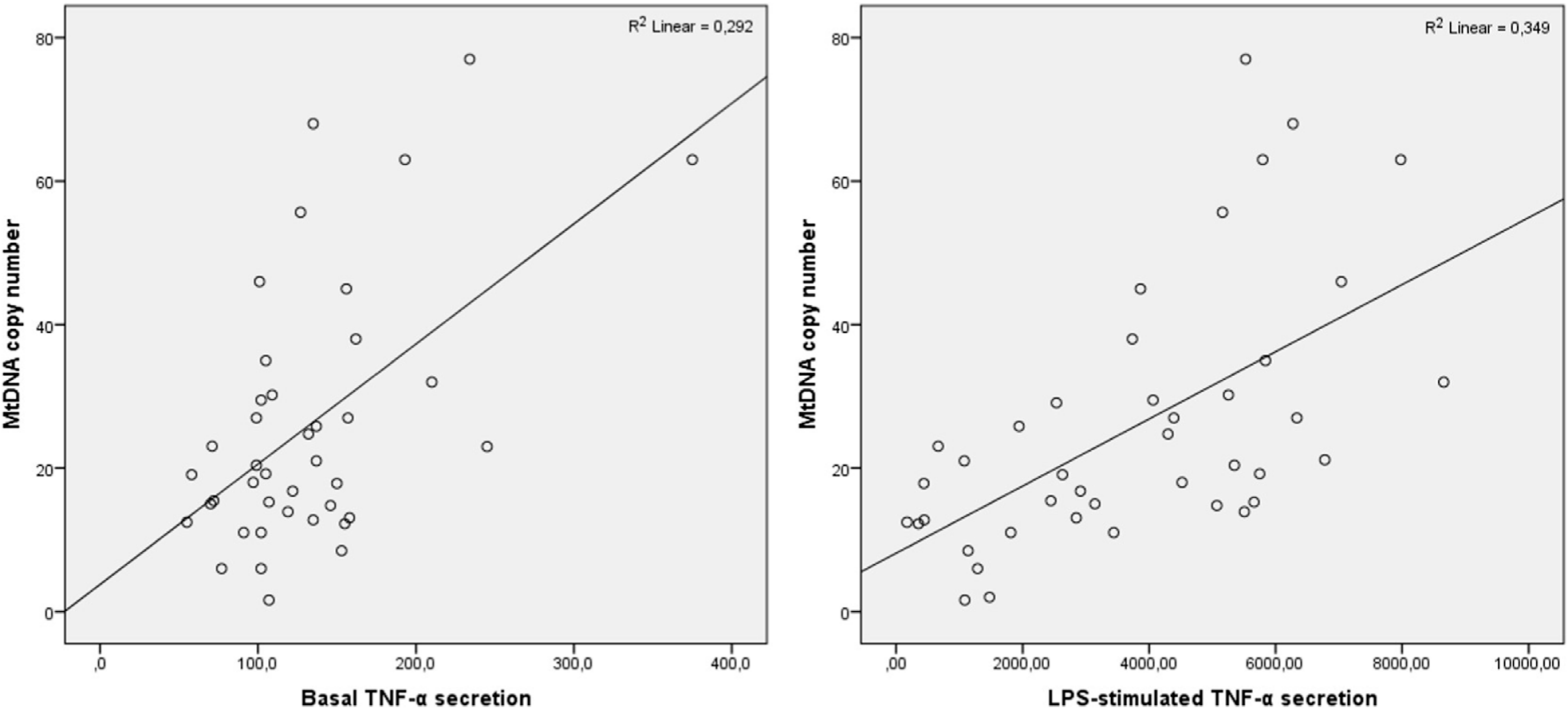
Correlation of TNF-α secretion and mtDNA copy number in patients with CHD and obesity. Pearson correlation coefficients are presented.

Pearson correlation analysis was conducted to evaluate the association of mtDNA copy number in CD14^+^ monocytes with basal and LPS-stimulated TNF-α secretion. It was demonstrated in total group that mtDNA copy number positively correlated with basal TNF-α secretion (R = 0.419, р = 0.002) as well as with LPS-stimulated secretion (R = 0.562, р<0.001), and TNF-α secretion after the second LPS-stimulation (R = 0.458, р = 0.001). The correlation analysis was performed in CHD, obesity and CHD + obesity groups. In the obesity group, mtDNA copy number correlated significantly only with LPS-stimulated TNF-α secretion (R = 0.585, р = 0.028). In the CHD group, mtDNA copy number correlated significantly with basal and LPS-stimulated TNF-α secretion (R = 0.892, р<0.001 and R = 0.725, р = 0.027, respectively). In the group of patients with CHD and obesity, mtDNA copy number correlated with basal TNF-α secretion (R = 0.465, р = 0.001) as well as with LPS-stimulated secretion (R = 0.591, р<0.001), and TNF-α secretion after the second LPS-stimulation (R = 0.353, р = 0.024). The results are presented at Figure 2.

## Discussion

The results of the current study demonstrated the statistically significant increase of both basal and LPS-stimulated TNF-α secretion by cultured monocytes of participants with CHD and obesity, that indicates the pro-inflammatory activation of circulating monocytes in obese patients with CHD (Hotamisligil et al., 1993). The measurements of TNF-α secretion in normal weight patients with CHD and in CHD-free obese study participants showed the increase of basal and LPS-stimulated TNF-α secretion in these groups, but this difference was not statistically significant in comparison with control group. The response to the second LPS stimulation was also significantly higher only in patients with obesity and CHD than in the control group. However, the immune response of monocytes to the second inflammatory stimulation was significantly lower than after the first LPS stimulation and was the similar to the level of basal secretion in all studied groups, that indicates the tolerance of the immune response of monocytes in terms of TNF-α. In general, TNF-α levels may be elevated in obesity and CHD due to disturbances in inflammatory and immune processes, as well as metabolic dysfunction and heart damage. Hypertrophy of adipocytes in obesity leads to the development of hypoxia promoting the activation of inflammatory mediators including monocyte chemotactic protein-1 that attracts immune cells and macrophages in adipose tissue (Litwin and Kułaga, 2021). It was shown that in obesity conditions adipocytes are the main producers of transmembrane TNF-α while soluble form of TNF- α is mostly secreted macrophages. Clinical studies demonstrated that serum levels of TNF-α are elevated in patients with obesity, and decrease with weight loss (Johnston and Abbott, 2023). These data indicate that macrophages play an important role in the development of inflammation in adipose tissue by producing chemokines and pro-inflammatory mediators including TNF-α, that contributes to maintaining the inflammatory response in tissues (Rogacev et al., 2010). At the same time, the disrupted blood supply in the heart in CHD conditions causes metabolic dysfunction followed activation the inflammatory mechanisms resulting in increase of the TNF-α production (Darenskaya et al., 2021).

Mitochondrial dysfunction is currently considered as one of the possible mechanisms of violation of the inflammatory response in the cell (Myakala et al., 2023). Quantitative determination of the mtDNA copy number is a conditional indicator of the content of mitochondria in a cell. This criterion is increasingly used as a biomarker of mitochondrial function that reflects the extent of mtDNA damage (Longchamps et al., 2020). Several studies show that changes of mtDNA copy number may be associated with various diseases, including cardiovascular disease (Castellani et al., 2020). However, existing data do not provide a direct understanding of the pathogenetic mechanisms of association between mtDNA levels and development of associated diseases.

It has been shown in the current study that patients with obesity and CHD and normal weight patients with CHD have significantly increased mtDNA copy number in comparison with control group. The increase in the number of copies of mitochondrial DNA in CHD may be due to various factors. It can be hypothesized that CHD-induced inflammation is accompanied with increased production of free radicals and oxidative stress, which causes changes in mitochondrial biogenesis (Bhatti et al., 2017). Violation of mitochondrial dynamics, namely, the division process, leads to an incomplete process, which results in an increase in the mitochondrial mass and, accordingly, mtDNA copy number. At the same time, the results of the recent study indicate that reduced level of mtDNA in whole blood was a risk factor for heart failure (Hong et al., 2020). It is assumed that mtDNA contains the genetic information necessary for the synthesis of proteins responsible for the function of mitochondria. A decrease in the level of mtDNA leads to a disruption in the synthesis of such proteins, that lead to the appearance of defective mitochondria (Sazonova et al., 2021). The results of our study match the data of the other study on the relationship between the increased BMI and mtDNA copies in buffy coat cells obtained from peripheral blood which demonstrated no association of slight increase in BMI with a change of the mtDNA copy number, while the significant increase of BMI is associated with increased mtDNA copy number (Skuratovskaia et al., 2019). On the other hand, studies of mtDNA from whole blood show results reporting that overweight patients have reduced mtDNA copy number (Bordoni et al., 2022). This finding is explained by the fact that obese patients often have elevated levels of oxidative stress. Excessive fat accumulation can lead to increased production of reactive oxygen species, which damage DNA, including mitochondrial DNA, followed the reduction of mtDNA copies (Lefranc et al., 2018). However, the results of the current study allow to suggest that the increase in mtDNA copies may be a compensatory mechanism that is implemented to maintain the optimal level of adenosine triphosphate production.

The most patients with obesity and CHD administrated statins, that could affect the inflammatory status of monocytes. The anti-inflammatory properties of statins have been confirmed in several studies (Satny et al., 2021). However, in clinical trials, different and inconsistent results have been obtained regarding the effect of statins on production of inflammatory markers. It was demonstrated in a systematic review that statin use did not affect serum concentrations of various inflammatory mediators including TNF-α, IL-6, sICAM and sVCAM (He et al., 2023). At the same time, another meta-analysis of randomized clinical trials aimed to determine the effect of statins on serum levels of TNF-α, MCP-1, VCAM1 and IL-6 in patients with cardiovascular diseases showed that statins have a beneficial effect on reducing the serum levels of TNF-α (Abbasifard et al., 2022). This highlights the complexity and diversity of the effects of statins on inflammatory processes in the body, and requires further research to fully understand their effects on inflammatory mediators.

Another important factor that can affect the inflammatory status of immune cells and mitochondrial functions is aging (Miwa et al., 2022). The results of the study conducted to investigate the relationship between the blood levels of inflammatory cytokines and increase in the number of mtDNA copies in the elderly demonstrated that patients with the highest mtDNA levels had increased levels of TNF-α, IL-6, RANTES and IL-1ra secretion in blood plasma, and conversely, patients with the lowest mtDNA levels had decreased levels of secretion of the same cytokines (Pinti et al., 2014). Regarding the association of aging, mitochondrial dysfunction with obesity and CVD, it is widely known that the metabolic changes associated with obesity are similar to those observed with aging. For example, obesity and aging have a similar set of phenotypes, such as mitochondrial dysfunction, genome changes, accumulation of intracellular macromolecules, and systemic inflammation (Nunan et al., 2022). In addition, mitochondrial dysfunction is directly related to chronic inflammation and oxidative stress, which affect the cardiovascular system. Thus, it can be concluded that aging, mitochondrial dysfunction, cardiovascular diseases and obesity are closely related.

An important feature of this experiment is the fact that mtDNA was isolated from CD14^+^ monocytes, that allowed investigating the relationship of mtDNA copy number with inflammatory response of cultured cells in terms of TNF-α. The direct association of changes in the number of mtDNA copies and the secretion of TNF-α by monocytes in individuals with CHD and obesity was revealed in the current study. Thus, the change in mtDNA copy number in CD14^+^ monocytes which indicates the presence of mitochondrial dysfunction, confirm the direct involvement of mitochondria in the violation of the inflammatory response of monocytes. The study was a pilot project aimed to evaluate the relationship of mitochondria number considered as an indicator of mitochondrial dysfunction and inflammatory status of monocytes assessed by secretion of inflammatory cytokine TNF-α in primary culture of monocytes under inflammatory stimulation in patients with CHD and obesity, so the number of study participants was limited. However, the study results indicate the impact of increased mtDNA copy number on inflammatory activation of monocytes, which is expressed in excess TNF-α secretion, in patients with CHD and obesity so the further study investigating a number of parameters of mitochondrial functions on a larger cohort should be planned to evaluate the association of mitochondrial dysfunction and violation of inflammatory response of innate immune cells.

## Data Availability

The original contributions presented in the study are included in the article/Supplementary material, further inquiries can be directed to the corresponding author.
